# Generation of Induced Pluripotent Stem Cells from the Prairie Vole

**DOI:** 10.1371/journal.pone.0038119

**Published:** 2012-05-31

**Authors:** Devanand S. Manoli, Deepa Subramanyam, Catriona Carey, Erik Sudin, Julie A. Van Westerhuyzen, Karen L. Bales, Robert Blelloch, Nirao M. Shah

**Affiliations:** 1 Department of Anatomy, University of California San Francisco, San Francisco, California, United States of America; 2 Department of Psychiatry, University of California San Francisco, San Francisco, California, United States of America; 3 Department of Urology, University of California San Francisco, San Francisco, California, United States of America; 4 Center for Reproductive Sciences, University of California San Francisco, San Francisco, California, United States of America; 5 Department of Psychology, University of California Davis, Davis, California, United States of America; University of California, San Diego, United States of America

## Abstract

The vast majority of animals mate more or less promiscuously. A few mammals, including humans, utilize more restrained mating strategies that entail a longer term affiliation with a single mating partner. Such pair bonding mating strategies have been resistant to genetic analysis because of a lack of suitable model organisms. Prairie voles are small mouse-like rodents that form enduring pair bonds in the wild as well as in the laboratory, and consequently they have been used widely to study social bonding behavior. The lack of targeted genetic approaches in this species however has restricted the study of the molecular and neural circuit basis of pair bonds. As a first step in rendering the prairie vole amenable to reverse genetics, we have generated induced pluripotent stem cell (IPSC) lines from prairie vole fibroblasts using retroviral transduction of reprogramming factors. These IPSC lines display the cellular and molecular hallmarks of IPSC cells from other organisms, including mice and humans. Moreover, the prairie vole IPSC lines have pluripotent differentiation potential since they can give rise to all three germ layers in tissue culture and in vivo. These IPSC lines can now be used to develop conditions that facilitate homologous recombination and eventually the generation of prairie voles bearing targeted genetic modifications to study the molecular and neural basis of pair bond formation.

## Introduction

Most animals exhibit transient affiliative behaviors with other members of their species. In a few mammalian species, such interactions lead to the formation of enduring social attachments that, in humans, include pair bonds between mating partners, biparental care of young, and kinships based on family or shared interests [1]–[3]. The traditional genetic model organisms, including mice, zebrafish, fruitflies, and nematodes do not form social attachments, thereby precluding molecular genetic approaches to study these striking behaviors [4], [5]. By contrast, prairie voles (*Microtus ochrogaster*) exhibit many forms of social attachment that resemble those observed in humans [6]. These rodents form socially monogamous pair bonds between mating partners who also exhibit biparental care of young, incest avoidance, and frequent aggressive rejection of other mating partners. In addition, experimental separation of pair bonded individuals elicits physiological signs of stress and elevated anxiety-like behaviors [7].

The behavioral analogy between social attachments in prairie voles and humans appears to extend to the underlying regulatory mechanisms. The neuropeptide hormones oxytocin and vasopressin mediate pair bonding behaviors in voles, and these hormones have also been implicated in social attachment-type behaviors in humans [6], [8]–[15]. Thus, prairie voles provide a valuable model to study the molecular and neural circuit basis of pair bonding and other forms of social attachment. Reverse genetic approaches to modify genetic loci in a targeted fashion would greatly facilitate the study of the molecular and neural circuit basis of pair bonding and its associated affiliative behaviors in prairie voles.

Targeted genetic modification in mammalian systems requires the generation of germline competent pluripotent stem cells that can be stably maintained in tissue culture and engineered via homologous recombination [16]. Therefore, as an initial step towards developing reverse genetic engineering in prairie voles, we have employed a modified version of the four factor reprogramming paradigm to generate eleven IPSC lines that bear the cellular, molecular, and differentiation signature of germline competent stem cells [17]–[22]. These prairie vole IPSC (PVi) lines will greatly facilitate the development of targeted genetics in this model organism.

## Results

### Reprogramming prairie vole embryonic fibroblasts

**Figure 1:**
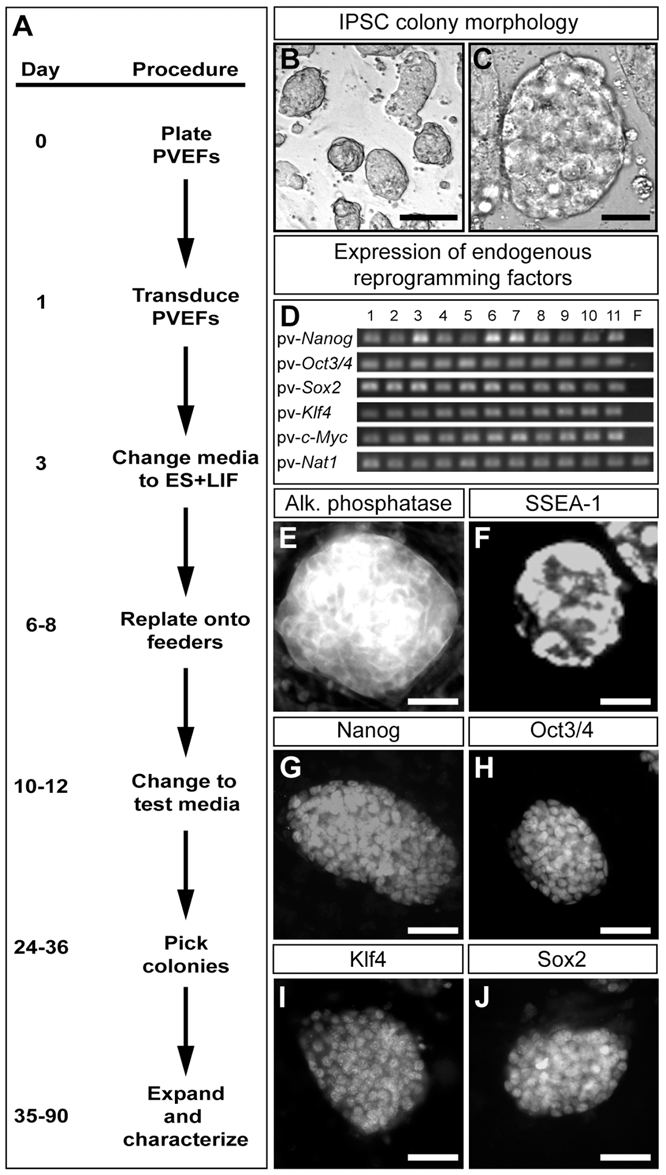
Induction and characterization of PVi lines. (*A*) Protocol for reprogramming PVEFs. All brightfield and immunolabeling images (B, C, E–J) depict colonies of a single representative PVi line (line 6). (*B–C*) Colony morphology of a PVi line. Colonies display morphology similar to that of mouse ES cells, including distinct raised colonies (B), with tightly-packed cells and well-defined, phase-bright margins (C). (*D*) RT-PCR for endogenous reprogramming factors in PVi lines. RT-PCR for pv-*Nat1* was performed as a positive control for an endogenous, ubiquitously expressed gene that should be expressed in all cells irrespective of their reprogramming state. Lanes 1–11 show PCR products from PVi lines 1–11, respectively. Lane 12 (“F”) shows PCR products from PVEFs. (*E*) PVi colony exhibiting alkaline phosphatase (Alk. phosphatase) activity. (*F*) Live immunofluorescent labeling of a PVi colony for SSEA-1. (*G–J*) Immunofluorescent labeling of PVi colonies for Nanog, Oct3/4, Klf4, and Sox2. Scale bars equal 500 µm (*B*), 100 µm (*C, E*), and 50 µm (*F–J*).

We obtained prairie vole embryonic fibroblasts (PVEFs) from gestation day 12–14 embryos using procedures previously used in the mouse [23]. Reprogramming of PVEFs was initiated by viral transduction of the four pluripotency-inducing transgenes (human orthologs of *Oct3/4*, *Sox2*, *Klf4*, and *c-Myc*; Figure 1A) [20], [22]. Although c-Myc enhances reprogramming in tissue culture, it also increases the rate of tumorigenicity in chimeric animals generated from IPSCs; in some experiments therefore, we also employed a three factor (*Oct3/4*, *Sox2*, *Klf4*) reprogramming protocol to determine if this would also yield IPSCs [21], [24]. PVEFs did not express the receptor (Slc7a1) required for infection with the ecotropic retrovirus used to transduce cells with the reprogramming factors. To enable transgene delivery in PVEFs, we therefore infected these cells first with an amphotropic lentivirus encoding Slc7a1 followed by infection with the ecotropic retroviruses separately encoding each of the reprogramming transgenes [20]. Expression of these reprogramming factors confers pluripotency on every cell type obtained from lab mice under conditions that are used for ES cell culture [25]–[27]. Although we observed colony formation from PVEFs under these conditions, further characterization (see below) revealed that none of these colonies contained pluripotent stem cells. We reasoned that such failure could result from poor expression of Slc7a1 in PVEFs, which would reduce the probability of transducing single cells with each of the reprogramming factor-encoding retroviruses required to induce pluripotency. We therefore used high titer amphotropic retroviruses to transduce PVEFs with the reprogramming transgenes [28]. Although we observed >10-fold more colonies with this viral transduction protocol, these colonies also did not contain any pluripotent stem cells. Of 1500 colonies analyzed in standard ES culture media, none yielded IPSCs (Table 1). These results indicate that pluripotency-inducing genes elicit only partial reprogramming of PVEFs grown in mouse ES cell culture media.

**Table 1:** Identification of culture conditions that promote generation of PVi lines.

Culture conditions	Colonies	Colonies picked	PVi lines
FBS+LIF	Yes	1025	0
FBS+LIF+5-aza-cytidine*	No		
FBS+LIF+MAPK/ERK inhibitor PD98059*	No		
FBS+LIF+ROCK inhibitor Y27632*	No		
FBS+LIF+bFGF+Activin A	No		
FBS+LIF+3iM	Yes	275	0
FBS+LIF+3iR	Yes	200	0
KSR+LIF	Yes	500	3
KSR+LIF+bFGF+Activin A	No		
KSR+LIF+3iM	Yes	400	1
KSR+LIF+3iR	Yes	400	7
***Total***		**2800**	**11**

Successful reprogramming of PVEFs into PVi lines was observed when FBS was replaced with KSR. For constituents of the 3iM and 3iR cocktails, which modulate distinct signal transduction pathways, please see Materials and Methods.

* , these supplements were either cell lethal or prevented colony formation.

Many tissue culture media supplements have been reported to enhance reprogramming induced by Oct3/4, Sox2, and Klf4 [29]–[37]. Because none of the colonies obtained from PVEFs grown in standard media yielded IPSCs following transduction with these reprogramming factors, we also tested such tissue culture supplements in an attempt to induce IPSCs (Figure 1A). None of these supplements to standard ES cell culture media yielded IPSCs (Table 1). In fact, many of these supplements were cell-lethal and did not even elicit colony formation from PVEFs. Standard ES media contains fetal bovine serum (FBS) and a recent commercially available serum replacement (knockout serum replacement, KSR) has been shown to enhance reprogramming when substituted for FBS in culture media [31]. We therefore cultured virally transduced PVEFs in KSR containing culture media in the presence or absence of various non-toxic supplements. Most of these conditions yielded colonies that contained pluripotent stem cells (Table 1). The media supplements 3iM and 3iR (see Methods for ingredients of 3iM and 3iR) enhance reprogramming in mice and rats, respectively. However, neither supplement increased the number of colonies obtained from PVEFs bearing the reprogramming transgenes (Table S1). Nevertheless, culture medium supplemented with 3iM or 3iR did yield bona fide IPSCs, with 3iR leading to a slight increase in the number of lines compared to medium containing 3iM (p<0.03, Chi-squared test; Table 1). In summary, of 1300 colonies analyzed in KSR-containing media, 11 yielded IPSC lines that fulfilled the standard cellular and molecular criteria for pluripotency. Importantly, these 11 IPSC lines (PVi1-11) were generated from three independent preparations of PVEF cells that were obtained from distinct prairie vole breeding pairs, with individual PVEF preparations yielding 2, 4, and 5 IPSC lines. Moreover a majority (3 of 4) of such PVEF preparations yielded IPSC lines, indicating that PVEFs represent a reliable source of reprogrammable cells in prairie voles.

### Characterization of colonies obtained from reprogrammed PVEFs

We first observed cellular aggregates 2–3 weeks subsequent to viral transduction of pluripotency-inducing transgenes into PVEFS. Many of these aggregates resulted in large phase bright colonies by 4–5 weeks, and we picked these for further expansion (Figure 1A–C). In general, colonies obtained in FBS-containing media usually had a phase-bright cobblestone appearance and their boundaries were often not sharply demarcated from the feeder cells. By contrast, KSR-containing media promoted the growth of colonies that resembled mouse ES colonies such that they had a smooth phase-bright appearance and a sharp border that clearly distinguished them from the underlying feeder cells (Figure 1B, C). We expanded all colonies such that they could eventually populate individual wells (9.5 cm^2^ surface area) of a 6-well plate. At this point, a subset of the cells within each well was frozen and the remainder was subjected to molecular characterization of the reprogrammed state. We tested each cell line for molecular hallmarks of pluripotency in a predetermined sequence such that colonies that did not meet a criterion were not analyzed further [17], [30]. We first tested colonies for expression of Nanog, an essential signature of the pluripotent state, by RT-PCR (Figure 1D) [38]. Persistent expression of the virally transduced reprogramming factors precludes subsequent differentiation and contribution to chimeric animals [18], [39]. We therefore utilized RT-PCR to identify the Nanog+ colonies that had switched off expression of these transgenes (Figure S1). Pluripotent colonies that silence expression of reprogramming transgenes maintain their pluripotent state by switching on expression of endogenous Oct3/4, Sox2, Klf4, c-Myc, and other molecular markers [17], [18]. Given that the prairie vole genome has not been sequenced yet, we verified endogenous expression of these genes using prairie vole-specific PCR primers (Figure 1D). These results have subsequently been validated using RT-quantitative PCR experiments, which show that each of these genes is expressed at levels significantly exceeding those observed in the parental PVEFs (Figure S2). Colonies were subsequently screened for alkaline phosphatase activity with a fluorescent substrate and for expression of SSEA-1 by immunolabeling (Figure 1E, F) [19]. Although expression of alkaline phosphatase or SSEA-1 is not exclusive to the pluripotent state, the cellular assays used to detect these markers afford a sensitive means to detect any heterogeneity within reprogrammed colonies. All IPSC lines were also screened for homogeneous expression of Nanog, Oct3/4, Klf4, and Sox2 using immunolabeling (Figure 1G–J). Our final criteria were formulated by practical considerations. Any IPSC line that will be used for gene targeting must have the capacity for expansion for several generations in tissue culture and the ability to be thawed from frozen stocks for additional manipulations. Each of our validated IPSC lines displays these traits and has been expanded for >8 generations in tissue culture. Cell populations from pluripotent stem cell lines eventually consist of many cells that are aneuploid as a consequence of repeated passage in tissue culture [40]–[42], and we have therefore maintained a frozen stock of our validated IPSC lines at low passage numbers (passage numbers 6–8). Extensive aneuploidy in ES or IPSC lines precludes efficient contribution to the chimera and germline [41], [43]–[45]. We therefore karyotyped our vole IPSC lines and determined that ≥70% of cells within each line were euploid (Table S2; 1n = 27 in prairie voles), a degree of euploidy that exceeds the minimum required for chimera generation and germline transmission with mouse ES cells [41], [46]. In summary, these cellular and molecular criteria are indicative of the pluripotent nature of the 11 IPSC lines we have obtained by reprogramming fibroblasts from the prairie vole.

### Vole IPSC lines have pluripotent differentiation capacity in tissue culture and in vivo

**Figure 2:**
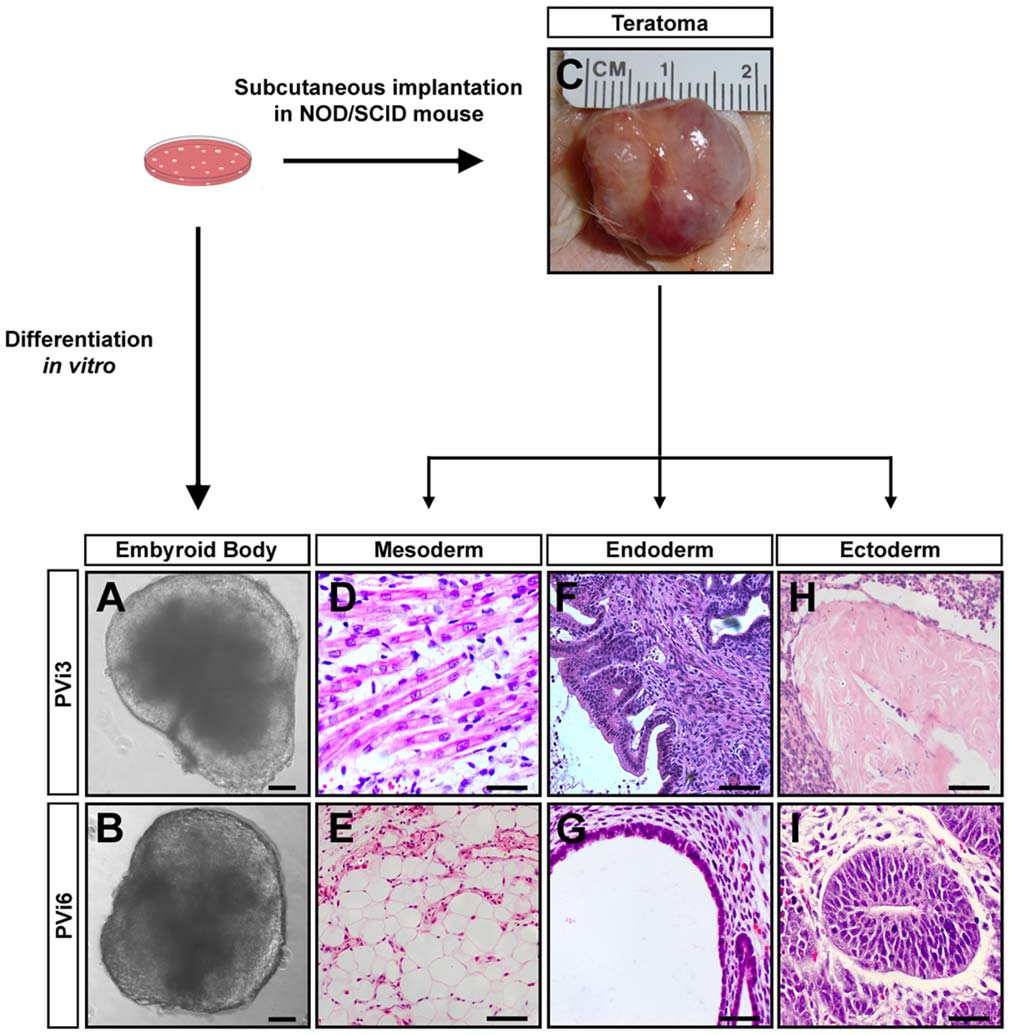
PVi lines are pluripotent in vitro and in vivo. (*A–B*) Differentiation of PVi lines in tissue culture yields embryoid bodies. (*C*) Teratoma obtained following subcutaneous implantation of PVi cells (PVi3) into a NOD/SCID mouse. (*D–I*) Hematoxylin and eosin stained tissue from teratomas obtained from PVi3 (D, F, H) and PVi6 (E, G, I) shows cellular differentiation into mesodermal, endodermal, and ectodermal lineages. Scale bars equal 100 µm (A, B, D–I).

We next wished to determine whether these reprogrammed prairie vole cells exhibit the potential to differentiate into cell types of all germ layers. Accordingly, we first tested the capacity of our vole cell lines to form embryoid bodies in tissue culture. We therefore performed suspension cultures of colonies from each reprogrammed prairie vole cell line that displayed the cellular and molecular characteristics of pluripotency. These colonies were grown in differentiation conditions until we observed the formation of spherical, often largely phase-dark, lobulated cysts (Figure 2A, B) that resembled mouse embryoid bodies. These cysts were tested for the expression of markers of all germ layers using RT-PCR. These studies revealed the expression of markers of the endoderm (Hnf4, Tie2, or Sox17), ectoderm (Sox1, Ker18, Pax6, or GFAP), and mesoderm (T, Gata4, AFP, or Flk-1) in each of 11 reprogrammed cell lines (Table 2). We also observed expression of Vasa, a marker of germ cell lineages [47], indicating that many of these lines form embryoid bodies with germ cell differentiation even in tissue culture.

**Table 2:** PVi lines are pluripotent in tissue culture.

EBs derived from	Lineage markers											
	Ectoderm				Endoderm			Mesoderm				Germ-line
	*Gfap*	*Ker18*	*Pax6*	*Sox1*	*Hnf4*	*Sox17*	*Tie2*	*Afp*	*Flk1*	*Gata4*	*T*	*Vasa*
PVi1	+		+		+	+		+	+		+	
PVi2		+	+	+	+	+				+	+	+
PVi3		+	+	+	+	+	+		+	+	+	+
PVi4		+	+	+	+	+	+		+	+	+	
PVi5	+	+	+	+		+				+	+	
PVi6	+		+	+	+	+	+	+		+		
PVi7		+	+	+	+	+			+	+	+	+
PVi8	+	+				+	+		+		+	
PVi9			+	+		+	+	+	+		+	
PVi10	+	+		+			+	+		+	+	
PVi11			+	+	+	+				+	+	

PVi lines generate embryoid bodies (EBs) that contain cell types representing all 3 somatic germ layers as revealed by RT-PCR for molecular markers of ectoderm, endoderm, and mesoderm. This analysis also shows that embryoid bodies from many PVi lines contain *Vasa*-expressing cells, thereby suggesting the presence of germ cells.

In order to determine whether reprogrammed vole cells could differentiate into all three germ layers in vivo, we determined their ability to form teratomas. We therefore injected 10^5^–10^6^ cells from individual lines subcutaneously into immunocompromised mice (NOD/SCID). Each of the 11 lines that generated embryoid bodies in vitro produced large, visible subcutaneous tumors within 3 weeks of implantation (Figure 2C). These tumors were dissected and analyzed for differentiation into various cell types using standard histological criteria. Tumors from all 11 lines contained a diverse array of differentiated cell types of all germ layers (Figure 2D–I), including mesoderm (skeletal muscle, smooth muscle, fat cells), ectoderm (keratin and neural rosettes), and endoderm (branched tubular formations resembling gut and other lumenal epithelial structures). Taken together, our findings demonstrate that we have reprogrammed prairie vole fibroblasts into cells that exhibit the cellular and molecular hallmarks of pluripotency and that have the capacity to differentiate into all major cell types in tissue culture and in vivo.

## Discussion

We report the generation of prairie vole pluripotent stem cell lines that exhibit the morphological and molecular hallmarks of pluripotent IPSCs from other species. These PVi lines are also functionally pluripotent since they can differentiate into all germ layers in tissue culture and in vivo. Some of the requirements for IPSC generation and propagation appear to be shared across different animals, including prairie voles. Thus, the standard reprogramming transgenes that have previously been shown to induce pluripotency in diverse cell types in various species were also effective in reprogramming prairie vole fibroblasts. In addition, the presence of LIF was also essential for the generation and maintenance of PVi lines. A surprising finding from our studies is that many of the previously described small molecule enhancers of reprogramming in other rodents either do not stimulate pluripotency in prairie vole fibroblasts or they are cell lethal. Moreover, we find that the presence of fetal bovine serum in culture medium inhibits the generation of pluripotent cells, which were observed only in media in which serum had been substituted with knockout serum replacement. Thus, prairie vole cells require a distinct set of culture conditions to enable reprogramming even in the presence of LIF and pluripotency-inducing transgenes.

Prairie voles exhibit social attachment such that mating partners are socially monogamous, prefer each other to strangers, and exhibit distress upon separation. Such pair bonded voles also exhibit biparental care of young, including alloparental care, and incest avoidance [6], [48]. These striking behaviors are observed not only in the laboratory setting, but they are also exhibited in the wild. It has been difficult to study the molecular and neural circuit mechanisms underlying these behaviors because of the absence of gene targeting in prairie voles. Moreover, such social attachment behaviors are not observed in mice or other traditional genetic model organisms. The PVi lines we have generated will greatly facilitate the development of gene modification by homologous recombination and the eventual generation of transgenic voles bearing targeted genetic manipulations. Previous work has established a large repertoire of genetic tools that permit a sophisticated understanding of the function of genes and cells in vivo in mice, and it should be possible to use these tools to characterize the basis for displays of social attachment.

Humans exhibit attachment behaviors at every level of social interaction and there appears to be a striking similarity in the molecular control of these behaviors between humans and prairie voles [6], [48], [13]. Oxytocin and vasopressin have been shown to influence social attachment in both species and future studies in transgenic voles should enable the mapping of neural circuits that respond to these neuropeptides. Disruption of social bonds is a common feature of many mental illnesses, and an understanding of the underlying neural circuits may permit novel therapeutic interventions [9], [10], [49], [50]. Few mammalian species exhibit social attachment behaviors between adults, which likely represent adaptive responses to unique ecological niches [51], [52]. In fact, many vole species such as montane and meadow voles do not exhibit pair bonding [6]. Using comparative genomics [53], [54], transgenesis [55], and targeted genetic manipulations with the prairie vole stem cells we report here, it will be possible to understand not only the mechanisms whereby social attachment is encoded in the brain, but also how evolution has shaped the underlying neural circuits to enable different behavioral outcomes in closely related species.

## Materials and Methods

### Isolation of embryonic fibroblasts

PVEFs were obtained from gestation day 12–14 embryos using procedures identical to those described for obtaining mouse embryonic fibroblasts (MEFs) [23]. Triturated tissue fragments from 3–4 embryos were plated in a T150 flask (Corning) in MEF medium: DMEM (4.5 g/L glucose) containing 15% FBS (Hyclone), 2 mM L-glutamine, 1× non-essential amino acids, 1× nucleosides, 1× 2-mercaptoethanol and 1× penicillin/streptomycin. This initial plating of cells and tissue fragments was cultured until confluent (3–4 days) and then dissociated into a single cell suspension with Trypsin/EDTA. These cells were frozen (1× freezing medium) and subsequently used for IPSC induction or they were expanded further by 1–2 passages, treated with Mitomycin C (Sigma; or irradiated) to induce cell cycle arrest, and frozen for later use as feeder cells. We also prepared feeder cells from MEFs using this protocol. All media and supplements were from Millipore except when otherwise noted. Animals were handled and maintained in accordance with IACUC protocols at UC San Francisco and UC Davis.

### IPSC induction with reprogramming transgenes delivered by ecotropic retroviruses

Lentivirus encoding mouse Slc7a1 receptor was generated by using Fugene (Roche) to transfect HEK293T cells (ATCC) with pMD.G, p8.91, and pLenti6/UbC-Slc7a1 as described previously [20]. In brief, the cells were cultured in DMEM containing 10% FBS and 1× penicillin/streptomycin and the medium was changed every day. Supernatant was collected from the cells 48 and 72 hours following transfection, pooled, passed through a 0.45 µm filter (Corning), and used to transduce PVEFs. Ecotropic retroviruses encoding *Egfp*, and the human orthologs of *c-Myc*, *Klf4*, *Oct3/4*, and *Sox2* were generated in PLAT-E packaging cells (gift from Dr. Shinya Yamanaka lab) as described previously [22]. PLAT-E cells were plated at 8×10^6^ cells/10 cm dish in the same medium as HEK293T cells, and they were transfected the next day with plasmids bearing Egfp or the individual reprogramming transgenes (pMXs vectors, Addgene) using Fugene. Supernatant was collected as described above. To initiate reprogramming, PVEFs were plated at 8×10^5^ cells/10 cm dish in MEF medium and infected the next day with Slc7a1-encoding lentivirus supernatant supplemented with 4 µg/mL polybrene (Sigma). These cells were trypsinized 48 hours later and replated at the original density on a 10 cm dish containing growth arrested feeders (PVEFs or MEFs). Equal volumes of supernatants containing each of the 5 retroviruses were mixed, supplemented with 4 µg/ml polybrene, and transferred to the dishes containing PVEFs. Following an overnight incubation in these supernatants, the medium was replaced with ES medium (MEF medium supplemented with 5% FBS and 1000 U/mL LIF) and changed daily until colonies were picked. In some experiments, FBS was replaced by 15% KSR (Invitrogen) in the ES medium.

### IPSC induction with reprogramming transgenes delivered by amphotropic retroviruses

PVEFs were plated in MEF medium at 3×10^4^ cells/well of a 6-well plate on 0.2% gelatin 1 day prior to transduction. PVEFs were infected with amphotropic retroviruses encoding *Venus Egfp* (6×10^8^ IU/mL) and the human orthologs of *Oct3/4* (4×10^8^ IU/mL), *Sox2* (1×10^8^ IU/mL), *Klf4* (4×10^8^ IU/mL), *c-Myc* (2×10^8^ IU/mL) (packaged by Harvard Gene Therapy Initiative) such that the culture medium contained 1 µL/mL of each retrovirus and 4 µg/mL polybrene [28]. The transduced cells were fed fresh MEF medium each day for 2 days after viral transduction, following which the medium was replaced with ES medium. The cells were also provided with fresh ES medium every 24–48 hours.

### Media supplements

The cocktail 3iM [37] contained inhibitors to GSK3ß (3 µM; CHIR99021, Stemgent) and MEK (0.8 µM; PD184352, Santa Cruz), and an FGF receptor antagonist (100 nM; PD173074, Stemgent). The cocktail 3iR [35] contained inhibitors to GSK3ß (3 µM; CHIR99021, Stemgent) and ERK (1 µM; PD0325901 Stemgent), and a TGFß type I receptor antagonist (0.5 µM; A-83-01, Stemgent). Basic FGF (Invitrogen) and activin-A (Invitrogen) were used at 20 ng/mL each [56].

### RNA isolation and PCR

Total RNA was isolated with the RNeasy Mini Kit (Qiagen) according to the manufacturer's protocol. All RNA samples were treated with DNase I (Amplification grade, Invitrogen) and reverse transcribed into cDNA using Superscript III (Invitrogen) and oligo-dT primers according to the manufacturer's protocol. qPCR reactions was performed either on an ABI Prism 7100 or an ABI 7900HT (Applied Biosystems). All primers used are listed in Table S3.

### Immunolabeling and staining for alkaline phosphatase

Cells for immunolabeling were grown on glass coverslips, rinsed with D-PBS, fixed at room temperature for 10 min in ice-cold 4% paraformaldehyde (PFA), and rinsed again with D-PBS. For immunolabeling, the cells were incubated in block buffer (D-PBS, 5% donkey serum, 0.1% Triton X-100) for 1 hour at room temperature. The cells were exposed to primary antibody in labeling buffer (D-PBS, 0.5% donkey serum, 0.1% Triton X-100) overnight at 4°C, rinsed 3 times in labeling buffer at room temperature, and incubated in labeling buffer containing the fluorophore-conjugated secondary antibody for 1 hour at room temperature. The cells were rinsed several times and the coverslips mounted on glass slides using Vectashield (Vector). The primary antibodies used were polyclonal rabbit anti-Oct3/4 (Santa Cruz, 1∶50), anti-Klf4 (Santa Cruz, 1∶100), anti-Sox2 (Millipore, 1∶1000), anti-Nanog (Abcam, 1∶60), and mouse anti-SSEA1 (DSHB, 1∶100). The secondary antibodies used were Cy3-conjugated donkey anti-rabbit (Jackson, 1∶800) and Alexa 488-conjugated donkey anti-mouse (Molecular Probes, 1∶300). Alkaline phosphatase staining was performed on fixed cells using the Vector Red Alkaline Phosphatase Substrate Kit I (Vector Labs) according to manufacturer's protocol.

### Karyotyping

Cells were grown to 70% confluence, trypsinized, and incubated in 0.56% KCl at 37°C for 10 min. Cells were rinsed in 3∶1 ice-cold methanol∶glacial acetic acid 3 times, and dropped on to glass slides to generate chromosome spreads. These spreads were stained with Leishmann's stain for 8 min, rinsed with water, cleared twice in xylene, and mounted in Depex (EMS). Chromosomes were enumerated from ≥15 cells with well-delineated spreads for each cell line.

### Embryoid body generation

IPSC lines grown to 80% confluence were trypsinized until the colonies detached. The tissue culture dish was flooded with MEF medium and the colonies were transferred to an ultra-low adherence dish (Corning) to promote differentiation into embryoid bodies. MEF medium was changed every 3–5 days until embryoid bodies were observed at 3–5 weeks.

### Teratoma generation

Vole IPSCs were injected subcutaneously into the flanks of NOD/SCID mice. Tumor nodules were removed after 4–6 weeks, fixed overnight in 4% PFA at 4°C, and embedded in paraffin. The samples were sectioned at 20 µm thickness and stained with hematoxylin and eosin.

This study was carried out in strict accordance with the recommendations in the Guide for the Care and Use of Laboratory Animals of the National Institutes of Health. The protocol was approved by the Institutional Animal Care and Use Committee of the University of California, San Francisco (Approval Number: AN081802-03B). All animals were sacrificed under carbon dioxide followed by decapitation, and all efforts were made to minimize suffering. All prairie vole cell lines (PVEFs and PVi) generated and used in this manuscript were generated from harvested tissue according to the protocol approved by the Institutional Animal Care and Use Committee of the University of California, San Francisco (Approval Number: AN081802-03B).

## Supporting Information

Figure S1 **PVi lines silence exogenous reprogramming factors.** RT-qPCR shows silencing of transduced reprogramming factors relative to that of an unsilenced line. All PVi lines show lower expression of exogenous *Oct3/4*, *Klf4*, and *c-Myc* that is statistically significant relative to the unsilenced line (p<0.05, Chi-squared test).
(DOC)

Figure S2 **PVi lines express endogenous markers of pluripotency.** (*A*–*E*) RT-qPCR shows expression of endogenous prairie vole (pv) *Nanog*, *Oct3/4*, *Sox2*, *Klf4*, and *c-Myc* in all PVi lines and minimal expression of these genes in the PVEF cells. Shown are fold changes in expression of each gene in the indicated cell line relative to the mean expression of the gene in all PVi lines. The mean expression of each gene in PVi lines vastly exceeded the expression level in PVEFs (pv-*Nanog*, 1.9×10^4^ fold; pv-*Oct3/4*, 2.1×10^4^ fold; pv-*Sox2*, 4.6×10^3^ fold; pv-*Klf4*, 1.8×10^3^ fold; pv-*c-Myc*, 1.2×10^3^ fold). Note that the expression of each gene was normalized to that of *GAPDH* and the data represent results from two technical replicates of RT-qPCR for each cell line.
(DOC)

Table S1 **Media supplements do not enhance colony formation from PVEFs.** The media supplements 3iM, 3iR, or FA (bFGF+Activin) do not increase the number of colonies formed from PVEFs compared to basal culture conditions in 15% FBS or 15% KSR. Numbers in parentheses indicate the number of PVi lines generated. All media contained LIF. Fold induction = (# colonies in media supplement)/(# colonies in basal conditions); n = 3 for each condition. OSK: viral transduction of Oct3/4, Sox2, and Klf4. OSKM: viral transduction of Oct3/4, Sox2, Klf4, and c-Myc.
(DOC)

Table S2 **Karyotype analysis of PVi lines.** Metaphase chromosome spreads from each PVi line were enumerated to quantitate the degree of euploidy (1n = 27).
(DOC)

Table S3 **Sequence of primers used for PCR.**
(DOC)
