# Supplementary material for: Generation of Induced Pluripotent Stem Cells from the Prairie Vole
Source: PLoS One. 2012 May 31;7(5):e38119. doi: 10.1371/journal.pone.0038119 (PMC3365000; doi:10.1371/journal.pone.0038119)
Supplement: Figure S1 — PVi lines silence exogenous reprogramming factors. RT-qPCR shows silencing of transduced reprogramming factors relative to that of an unsilenced line. All PVi lines show lower expression of exogenous Oct3/4, Klf4, and c-Myc that is statistically significant relative to the unsilenced line (p<0.05, Chi-squared test). (DOC) [file pone.0038119.s001.doc]

**SUPPORTING INFORMATION**

| 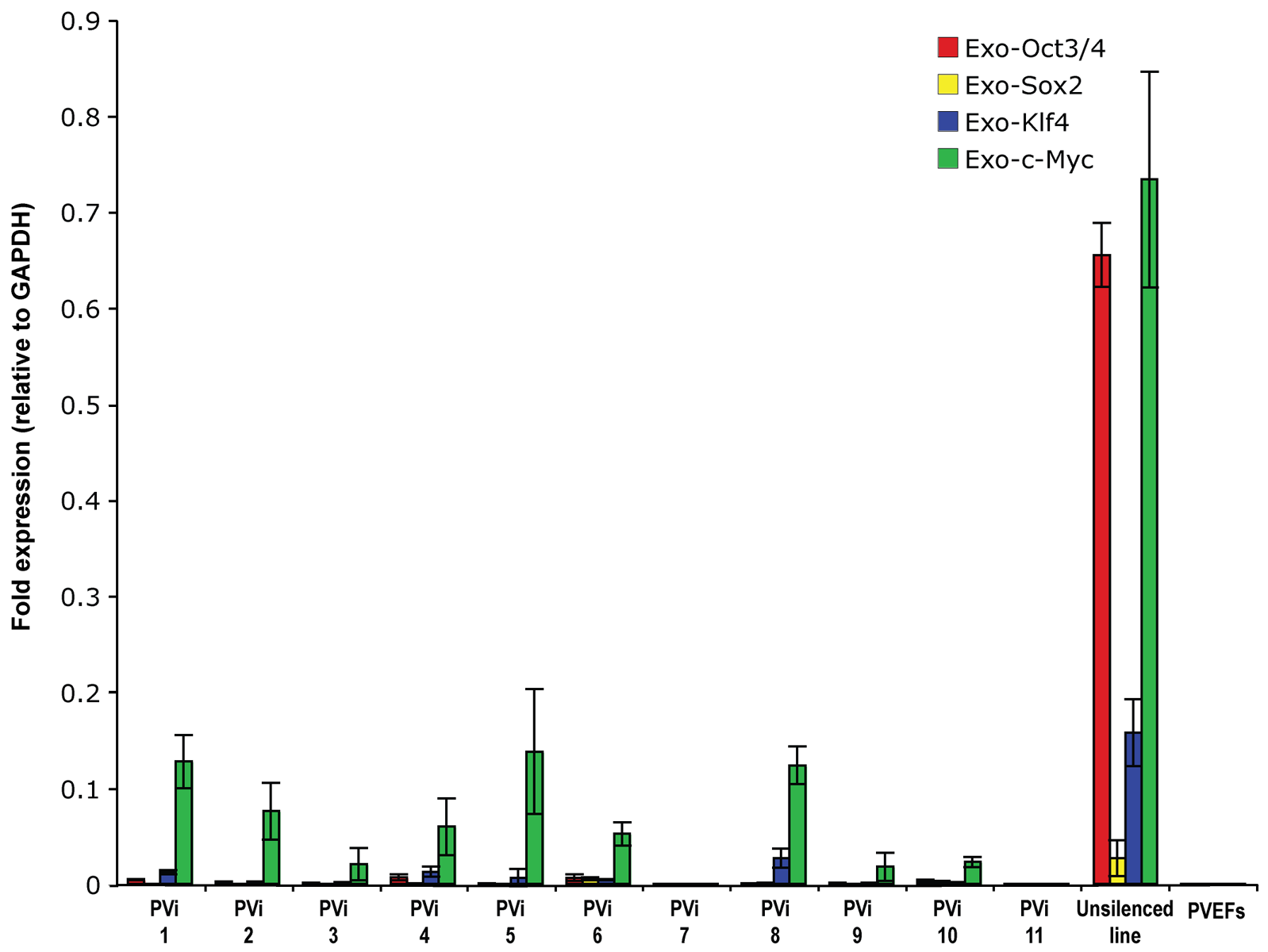 |
| --- |
| **Figure S1: PVi lines silence exogenous reprogramming factors**  RT-qPCR shows silencing of transduced reprogramming factors relative to that of an unsilenced line. All PVi lines show lower expression of exogenous Oct3/4, Klf4, and c-Myc that is statistically significant relative to the unsilenced line (p < 0.05, Chi-squared test). |
