# Supplementary material for: Generation of Induced Pluripotent Stem Cells from the Prairie Vole
Source: PLoS One. 2012 May 31;7(5):e38119. doi: 10.1371/journal.pone.0038119 (PMC3365000; doi:10.1371/journal.pone.0038119)
Supplement: Figure S2 — PVi lines express endogenous markers of pluripotency. (A–E) RT-qPCR shows expression of endogenous prairie vole (pv) Nanog, Oct3/4, Sox2, Klf4, and c-Myc in all PVi lines and minimal expression of these genes in the PVEF cells. Shown are fold changes in expression of each gene in the indicated cell line relative to the mean expression of the gene in all PVi lines. The mean expression of each gene in PVi lines vastly exceeded the expression level in PVEFs (pv-Nanog, 1.9×10^4^ fold; pv-Oct3/4, 2.1×10^4^ fold; pv-Sox2, 4.6×10^3^ fold; pv-Klf4, 1.8×10^3^ fold; pv-c-Myc, 1.2×10^3^ fold). Note that the expression of each gene was normalized to that of GAPDH and the data represent results from two technical replicates of RT-qPCR for each cell line. (DOC) [file pone.0038119.s002.doc]

**SUPPORTING INFORMATION**

| 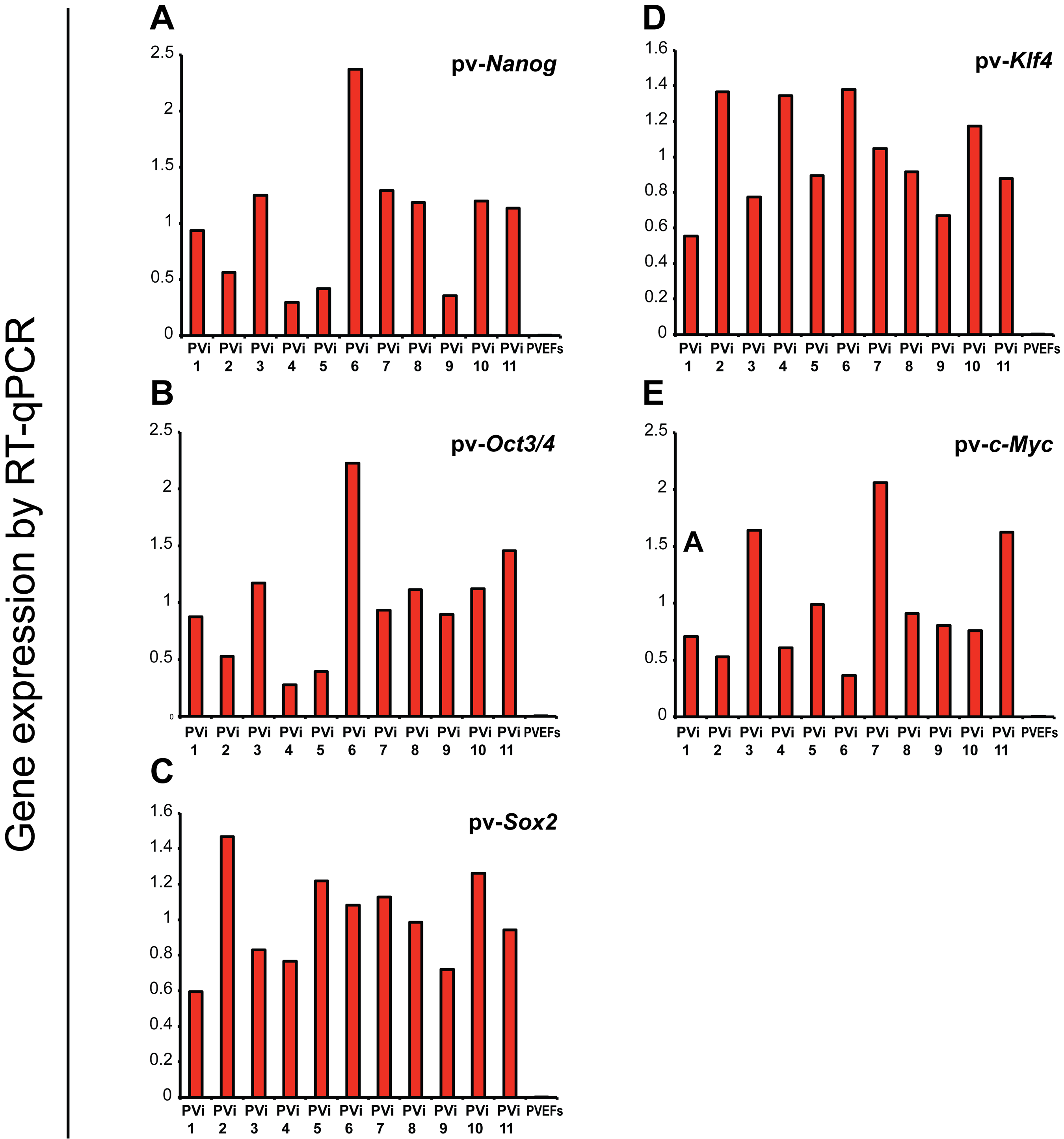 |
| --- |

**Figure S2: PVi lines express endogenous markers of pluripotency**

(*A*-*E)* RT-qPCR shows expression of endogenous prairie vole (pv) *Nanog*, *Oct3/4*, *Sox2*, *Klf4,* and *c-Myc* in all PVi lines and minimal expression of these genes in the PVEF cells. Shown are fold changes in expression of each gene in the indicated cell line relative to the mean expression of the gene in all PVi lines. The mean expression of each gene in PVi lines vastly exceeded the expression level in PVEFs (pv-*Nanog*, 1.9x10^4^ fold; pv-*Oct3/4*, 2.1x10^4^ fold; pv-*Sox2*, 4.6x10^3^ fold; pv-*Klf4*, 1.8x10^3^ fold; pv-*c-Myc*, 1.2x10^3^ fold). Note that the expression of each gene was normalized to that of *GAPDH* and the data represent results from two technical replicates of RT-qPCR for each cell line.
