# Supplementary material for: Generation of Induced Pluripotent Stem Cells from the Prairie Vole
Source: PLoS One. 2012 May 31;7(5):e38119. doi: 10.1371/journal.pone.0038119 (PMC3365000; doi:10.1371/journal.pone.0038119)
Supplement: Table S1 — Media supplements do not enhance colony formation from PVEFs. The media supplements 3iM, 3iR, or FA (bFGF+Activin) do not increase the number of colonies formed from PVEFs compared to basal culture conditions in 15% FBS or 15% KSR. Numbers in parentheses indicate the number of PVi lines generated. All media contained LIF. Fold induction = (# colonies in media supplement)/(# colonies in basal conditions); n = 3 for each condition. OSK: viral transduction of Oct3/4, Sox2, and Klf4. OSKM: viral transduction of Oct3/4, Sox2, Klf4, and c-Myc. (DOC) [file pone.0038119.s003.doc]

**Table S1: Media supplements do not enhance colony formation from PVEFs**

| **Basal induction conditions** | **Fold induction with different media supplements** | | | |
| --- | --- | --- | --- | --- |
|  | **No Supplement** | **3iM** | **3iR** | **FA** |
| OSK + FBS | 1 (0) | 0.5 (0) | 0.75 (0) | 0 (0) |
| OSK + KSR | 1 (0) | 1.33 (1) | 0.33 (0) | 0 (0) |
| OSKM + FBS | 1 (0) | 0.95 (0) | 1.16 (0) | 0.03 (0) |
| OSKM + KSR | 1 (3) | 1.02 (0) | 1.19 (7) | 0.24 (0) |

The media supplements 3iM, 3iR, or FA (bFGF + Activin) do not increase the number of colonies formed from PVEFs compared to basal culture conditions in 15% FBS or 15% KSR. Numbers in parentheses indicate the number of PVi lines generated. All media contained LIF.

Fold induction = (# colonies in media supplement)/(# colonies in basal conditions); n = 3 for each condition.

OSK: viral transduction of Oct3/4, Sox2, and Klf4.

OSKM: viral transduction of Oct3/4, Sox2, Klf4, and c-Myc.
