# Supplementary material for: Generation of Induced Pluripotent Stem Cells from the Prairie Vole
Source: PLoS One. 2012 May 31;7(5):e38119. doi: 10.1371/journal.pone.0038119 (PMC3365000; doi:10.1371/journal.pone.0038119)
Supplement: Table S2 — Karyotype analysis of PVi lines. Metaphase chromosome spreads from each PVi line were enumerated to quantitate the degree of euploidy (1n = 27). (DOC) [file pone.0038119.s004.doc]

**Table S2: Karyotype analysis of PVi lines**

| **PVi Line** | **% cells with**  **normal karyotype** |
| --- | --- |
| 1 | 75 |
| 2 | 86.7 |
| 3 | 73.3 |
| 4 | 80 |
| 5 | 93.3 |
| 6 | 80 |
| 7 | 80 |
| 8 | 93.3 |
| 9 | 86.7 |
| 10 | 85 |
| 11 | 80 |

Metaphase chromosome spreads from each PVi line were enumerated to quantitate the degree of euploidy (1n = 27).
