# Supplementary material for: Generation of Induced Pluripotent Stem Cells from the Prairie Vole
Source: PLoS One. 2012 May 31;7(5):e38119. doi: 10.1371/journal.pone.0038119 (PMC3365000; doi:10.1371/journal.pone.0038119)
Supplement: Table S3 — Sequence of primers used for PCR. (DOC) [file pone.0038119.s005.doc]

**Table S3: Sequence of primers used for PCR**

| **Gene** | **Forward primer** | **Reverse primer** |
| --- | --- | --- |
| *Brachyury(T)* | TACCCCAGCCCCTATGCTCA | GGCACTCCGAGGCTAGACCA |
| exogenous *Klf4* | CCTTACACATGAAGAGGCAC | CTTTTATTTTATCGTCGACC |
| exogenous *c-Myc* | CAGAGGAGGAACGAGCTGAA | CTTTTATTTTATCGTCGACC |
| exogenous *Oct3/4* | TCTCCCATGCATTCAAACTG | CTTTTATTTTATCGTCGACC |
| exogenous *Sox2* | CTGCCCCTGTCGCACATGTG | CTTTTATTTTATCGTCGACC |
| *Flk-1* | TCCTACAGACCCGGCCAAAC | ACACGTTGGCAGCTTGGATG |
| *GAPDH* | GCAATGCATCCTGCACCACCA | TTCCAGAGGGGCCATCCACA |
| *Gata4* | ACCAGAAAACGGAAGCCCAA | ATGGAGCTGCTGTGCCCATA |
| *Hnf4* | CACCAAGAGGTCCATGGTGTTTAAAG | CTTTGAGGCAGGCATATTCATTGTC |
| *Keratin 18* | CGCTTGCTGGAGGATGGA | CTTCTGCACAGTTTGCATGGA |
| *Nat1* | ATTCTTCGTTGTCAAGCCGCCAAAGTGGAG | AGTTGTTTGCTGCGGAGTTGTCATCTCGTC |
| *Pax-6* | AGTTCTTCGCAACCTGGCTA | AGTTGGTGTTCTCTCCCCCT |
| pv- *Klf4* | TCGGCGTCAGCTTCATCCTCGTCTTC | GCGCAGATTCTCGGCTGTAGAGGAG |
| pv- *c-Myc* | TGCGTGACCAGATCCCAGAGTTGG | ACTGTTCTCTCCGCTTCCTCAGT |
| pv- *Nanog* | GCAAGAACTTTCCAACATCCTGA | CCACATTGGAAGGTTTCCAGTCATGT |
| pv- *Oct3/4* | GAAGGGCAAGCAATCAAGCAGTG | TGCAGTGCAATGAGGGCTCCCATA |
| pv-*Sox2* | GACCTGATCAGCACGTACCTTAC | GTCGTTAATGGCCGTGGCGGGC |
| *Sox1* | GTCATGTCCGAGGCCGAGAA | AGCAGCGTCTTGGTCTTGCG |
| *Sox17* | AGCTCCAGAAACTGCAGACCAGAAG | CTCTTGGGGAAATAGGAAGGCTGAA |
| *Tie2（Tek)* | TTGGGGATGGACCCATCAAA | CAGTCCGCGGCTCCAAGTAG |
| *Vasa* | GACCTTCCTTCTACCATTGATGAGTAT | TCGCTCTGCCAGTATTTCCA |
